# Development of a quadruple qRT-PCR assay for simultaneous identification of hypervirulent and carbapenem-resistant *Klebsiella pneumoniae*

**DOI:** 10.1128/spectrum.00719-23

**Published:** 2023-12-07

**Authors:** Zhixiang Xu, Baisheng Li, Yushan Jiang, Jia Huang, Lebin Su, Weibo Wu, Qilin Pang, Zhuolin Li, Jiaqi Zhang, Xiaohe Li, Jun Wang, Fulan Cen, Ling Peng, Jinhu Liang, Fuxiang Wang, Chang Liu, Chenguang Shen, Yingxia Liu, Yang Yang

**Affiliations:** 1 Savid Medical School, University of Chinese Academy of Sciences, Beijing, China; 2 Shenzhen Key Laboratory of Pathogen and Immunity, Shenzhen Clinical Research Center for infectious disease, Shenzhen Third People’s Hospital, Second Hospital Affiliated to Southern University of Science and Technology, Shenzhen, Guangdong, China; 3 Guangdong Provincial Key Laboratory of Pathogen Detection for Emerging Infectious Disease Response, Guangdong Center for Disease Control and Prevention, Guangzhou, Guangdong, China; 4 BSL-3 Laboratory (Guangdong), Guangdong Provincial Key Laboratory of Tropical Disease Research, School of Public Health, Southern Medical University, Guangzhou, Guangdong, China; 5 Zhaoqing Center for Disease Control and Prevention, Zhaoqing, Guangdong, China; University of Pretoria, Pretoria, South Africa

**Keywords:** *Klebsiella pneumoniae*, hypervirulent, carbapenem-resistant, differential diagnosis, multiplex qRT-PCR

## Abstract

The increasing prevalence of hypervirulent (hv) and carbapenem-resistant (CR) *Klebsiella pneumoniae* (Kp) highlights the importance of timely and accurate differential diagnosis for epidemiological investigation and clinical management. A multiplex quantitative real-time PCR (qRT-PCR) assay for the simultaneous identification of hvKp and CR-Kp was developed and validated with excellent performance in sensitivity and specificity. Generally, the *gltA* gene for Kp, the *iucA*, *rmpA* and *rmpA2* genes for hvKp, and the *Klebsiella pneumoniae* carbapenemases (KPC) gene for CR-Kp were included in the qRT-PCR assay. The detection limits for classic Kp (cKp), CR-cKp, hvKp, and CR-hvKp strains could all reach 50 genome equivalent copies and 20 CFUs per reaction with high accuracy (*R* ^2^ > 0.99) and reliability (CV values < 3%). Detection results from 84 Kp positive clinical samples showed 31 hvKp with 8 CR-hvKp and 53 cKp with 1 CR-cKp strains. The presence of virulence-associated factors for the identified hvKp and KPC genes for CR-Kp was confirmed by previously developed conventional PCR and antimicrobial susceptibility tests, respectively. Furthermore, the qRT-PCR identified hvKp strains showed mortality rates of ≥40% in the outbred murine infection model, while no death for the identified cKp strains. These results indicated that our multiplex qRT-PCR assay could accurately identify hvKp and CR-Kp strains, which will be of great use for the rapid and accurate diagnosis in a clinical setting and the surveillance of the circulating Kp.

**IMPORTANCE:**

Globally, the increasing number of hypervirulent *Klebsiella pneumoniae* (hvKp) and carbapenem-resistant Kp (CR-Kp) infections poses a huge public health challenge with high morbidity and mortality. Worrisomely, due to the mobility of elements carrying virulence and drug-resistance genes, the increasing prevalence of CR-hvKp has also been found with an overwhelming mortality rate in recent years. However, the current detection methods for hvKp and CR-Kp have many disadvantages, such as long turnaround time, complex operation, low sensitivity, and specificity. Herein, a more sensitive, rapid, single-reaction, and multiplex quantitative real-time PCR was developed and validated to differentiate the circulating lineages of Kp with excellent performance in sensitivity and specificity, providing a useful tool for the differential diagnosis and the surveillance of the circulating Kp.

## INTRODUCTION

*Klebsiella pneumoniae* (Kp) is an increasingly important bacterial pathogen that is capable of causing severe organ and life-threatening diseases with two pathotypes termed classical Kp (cKp) and hypervirulent Kp (hvKp) presently circulating (1). The cKp is mainly an opportunistic pathogen causing bacteremia, urinary tract infection, and pneumonia in frequently healthcare-exposed or immunocompromised patients (2). The hvKp is characterized by hypermucoviscosity and unique capsular serotype, virulence gene, sequence type (ST), and resistant spectrum, which was first reported in Taiwan in 1986 (2 – 4). Moreover, hvKp could lead to disseminated invasive infections accompanied by liver abscesses, endophthalmitis, meningitis, and septic arthritis in otherwise healthy individuals (2 – 4). Patients infected with hvKp typically have a poor prognosis with a mortality rate of 3%–42% due to the rapid progression of infection requiring specific treatment such as vitrectomy and intravitreal antibiotics for hvKp-induced endophthalmitis (1, 5). Over the past few decades, an increasing number of hvKp infections have spread worldwide, with the highest prevalence in the Asian-Pacific region, posing a serious threat to the public (6). Recently, a multi-center study in China showed that the prevalence of hvKp infection ranged between 8.33% and 73.91% with a wide geographic distribution (7). Siderophore is an important virulence factor that scavenges scarce ferric iron from the environment (1). Total siderophore production dominated by aerobactin and increased capsule production mediated by *rmpA* or *rmpA2* have been shown to strongly correlate with the *in vivo* virulence of Kp (7 – 12). Therefore, *iucA* and/or either *rmpA* or *rmpA2* have been predicted to be the most accurate and durable molecular biomarkers for hvKp (1).

Carbapenems are a class of β-lactam antibiotics with broad-spectrum antibacterial activity serving as the first-line treatment for severe infections caused by multidrug-resistant Gram-negative Enterobacteriaceae, particularly extended-spectrum β-lactamase (13, 14). The appearance of carbapenem-resistant Enterobacteriaceae (CRE) strains has been increasingly reported with extensive clinical application, and about 70%–90% of CRE strains are carbapenem-resistant Kp (CR-Kp) in clinical practice (15 – 17). Globally, the increasing number of CR-Kp infections poses huge public health challenges with high morbidity and mortality (18, 19). The epidemiological analysis of CR-Kp in China showed that the resistance rate of Kp to imipenem increased significantly from 3% in 2005 to 25.3% in 2019 (20). In New York, the in-hospital mortality rate of patients infected with CR-Kp was 48%, higher than those infected with carbapenem-susceptible Kp (21). *Klebsiella pneumoniae* carbapenemases (KPC) is the most common carbapenemases produced by Kp, constituted by a new mutant of class A β-lactamase enzymes capable of hydrolyzing all known β-lactam antibiotics (22). To date, a total of 10 KPC gene variants from *bla_KPC-2_* to *bla_KPC-11_* have been found, and the main differences lie in some synonymous mutations (23).

Worrisomely, due to the mobility of elements carrying virulence and drug-resistance genes, the increasing prevalence of CR-hvKp has been found with overwhelming mortality in recent years (24). Hence, the rapid and accurate detection of hvKp and KPC-producing Kp in the clinical laboratory is essential to guide appropriate treatment and preventing dissemination. However, the current detection methods for hvKp and CR-Kp have many disadvantages, such as long turnaround time, complex operation, low sensitivity, and low specificity, which bring difficulties for early clinical diagnosis (25 – 30). Therefore, there is an urgent need to establish a simple, fast, and sensitive assay to accurately identify the phenotypes of the Kp strains. In this study, we established and validated a quadruple quantitative real-time PCR (qRT-PCR) assay for simultaneous identification of hvKp and CR-Kp.

## RESULTS

### Development of the multiplex qRT-PCR assay

All available sequences of *gltA*, *iucA, rmpA*, *rmpA2*, and KPC genes were retrieved from GenBank for alignment using MEGA 7.0. To obtain the specific primer/probe set for the assay development, we selected the highly conserved regions for primer/probe design using the Primer Premie 5.0 software (Table 1). The primer-probe set was optimized in a single multiplex qRT-PCR assay with a final concentration of 400 nM for each primer and probe. Four different phototypes of Kp (cKp, CR-cKp, hvKp, and CR-hvKp) confirmed by outbred murine infection model, string test, antimicrobial susceptibility test, and conventional PCR were used to test the specificity of the multiplex qRT-PCR assay. All four phenotypes of Kp were tested positive for the *gltA* gene (Table S1). Meanwhile, *iucA* and *rmpA/2* genes were positive for the two hvKp isolates, and the KPC gene was positive for the two CR-Kp isolates, with Ct values similar to the *gltA* gene. Moreover, there was also no non-specific amplification when testing other important respiratory pathogens such as influenza A virus (panH1N1, H3N2, and H9N2), influenza B virus (Victoria and Yamagata lineages), seasonal coronaviruses (HCoV-NL63, HCoV-OC43, and HCoV-229E), SARS-CoV-2, *Streptococcus pneumoniae, Staphylococcus aureus, Legionella pneumophilia, Haemophilus influenzae, Pseudomonas aeruginosa, Acinetobacter baumannii, Moraxella catarrhalis*, and *Mycobacterium tuberculosis*.

**TABLE 1:** Primers and probes used in the multiplex qRT-PCR assay

Primers and probes^*a*^	Sequences (5′−3′)^*b*^	Final concentration (nM)
*gtlA*-F	GAATTCAAAACTACCGTCACCCG	400
*gtlA*-R	TCACGTCGAGGGAATCATGATAGAA	400
*gtlA*-P	TCATGAGCAGATCACCCGTCTGTTCCA	400
*iucA*-F	TACTTCCCTTATTACCTGCTGGTTA	400
*iucA*-R	CATCAGATTCGCTTCACTGTCCA	400
*iucA*-P	CACTTTTGCCGTGACCGCCGCGCT	400
*rmpA/2-*F	ATAAGAGTATTGGTTGAYAGCMGGA	400
*rmpA/2-*R	GATGTCATAATCACACCCTTTAGGA	400
*rmpA/2-*P	CAATGGATGTGGCTTGACRTTTCGGG	400
KPC-F	ACCATTCGCTAAACTCGAACAGGAC	400
KPC-R	CTTGAATGAGCTGCACAGTGGGA	400
KPC-P	TGGCGGCTCCATCGGTGTGTACG	400

^*a*^ F: Forward primer; R: Reverse primer; P: Probe.

^*b*^ Y = T or C, R = A or G, M = A/C.

### Analytical sensitivity and reliability of the multiplex qRT-PCR assay

**Fig 1:**
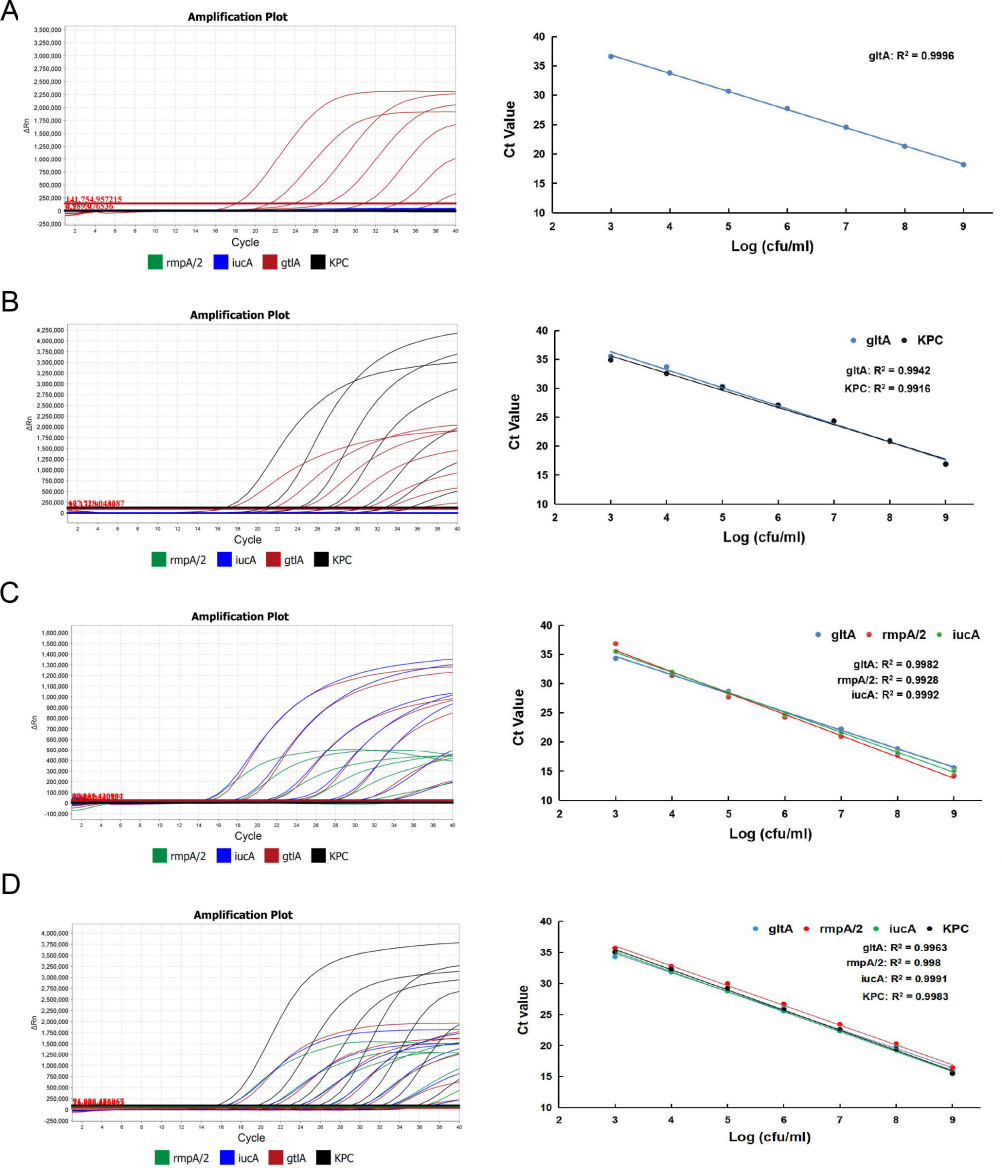
Sensitivity of the multiplex qRT-PCR assay using bacterial DNA of cKp, CR-cKp, hvKp, and CR-hvKp strains. (**A**) to (**D**) show the representative amplification plot and standard curves for 10-fold serial dilutions of bacterial DNA from cKp, CR-cKp, hvKp, and CR-hvKp, respectively.

**Fig 2:**
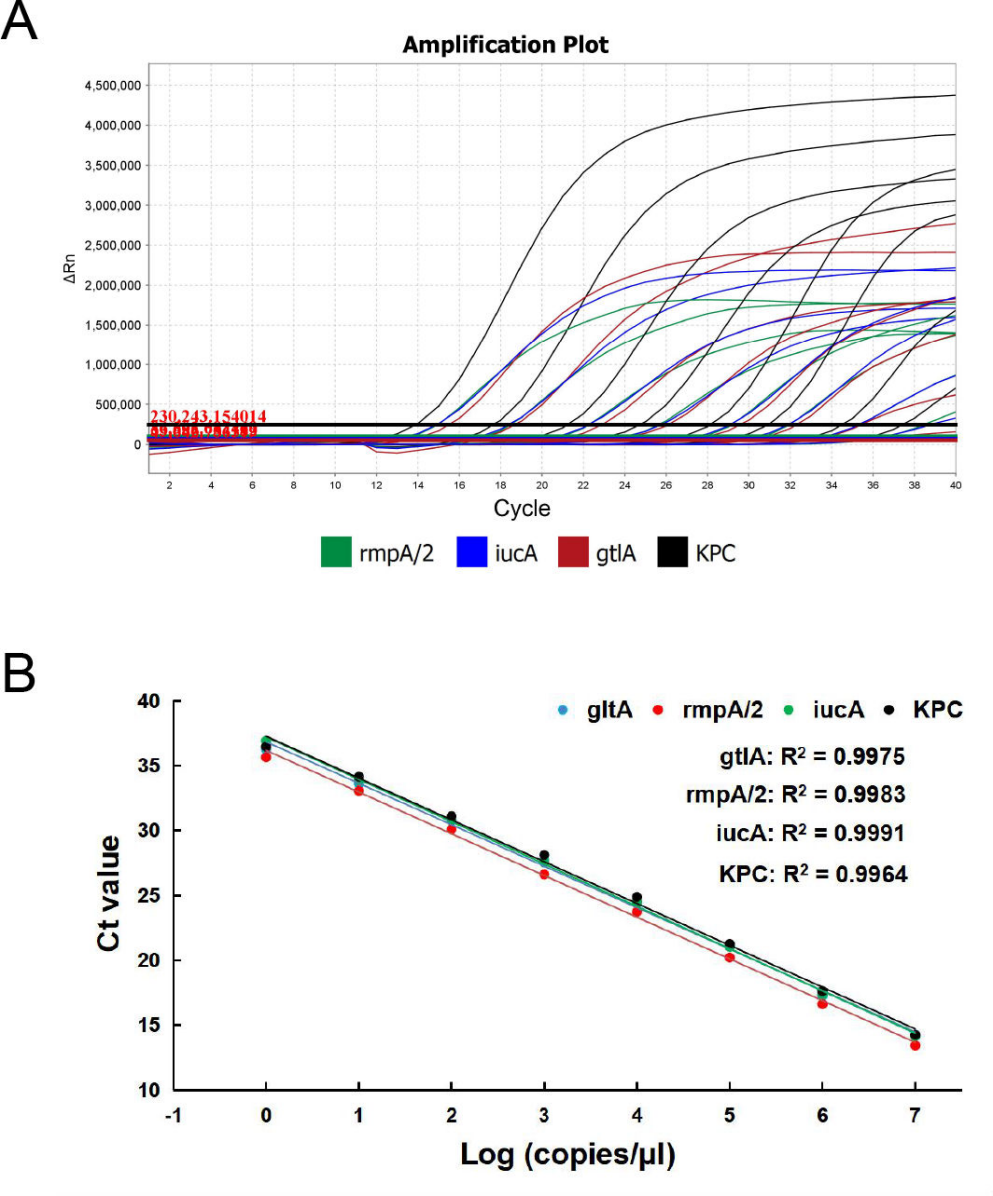
Sensitivity of the multiplex qRT-PCR assay using standard plasmid. (**A**) Representative amplification plot of the different concentrations of plasmid expressing the targeted *gltA*, *iucA*, *rmpA/2,* and KPC genes (10-fold dilutions). (**B**) Standard curve for 10-fold serial dilutions of the DNA standards. The log numbers of plasmid (copies/μL) are expressed linearly on the *x*-axis, whereas Ct values obtained from qRT-PCR are expressed linearly on the *y*-axis.

The limit of detection (LOD) for each target of the multiplex qRT-PCR assay was determined using serial 10-fold diluted standard plasmid and bacterial DNA. The amplification results from DNA standards ranging from 1 × 10^7^ to 1 × 10^1^ genome equivalent copies per milliliter were shown in Fig. 1. Linear cycle threshold (Ct) values were found among different concentrations of DNA standards with regression coefficients (*R* ^2^) >0.99 and similar Ct values among the four targets. Meanwhile, the LOD of the four targets reached 50 genome equivalent copies per reaction with typical amplification curves (Fig. 1). For the LOD of live bacteria, the bacterial DNA was extracted from serial 10-fold diluted overnight bacterial cultures at concentrations ranging from 1 × 10^9^ to 1 × 10^2^ CFU/mL (Fig. 2). The amplification results suggested that the obtained Ct values correlated well with serial diluted bacterial concentrations, with *R* ^2^ values above 0.99 for all the four targets. Moreover, the LOD of cKp, CR-cKp, hvKp, and CR-hvKp reached 20 CFUs per reaction. The high LOD suggest that our assay is sufficient for early clinical diagnosis.

The intra-assay and inter-assay variability were evaluated using the different concentrations of standard plasmid and bacterial DNA (Table 2). The intra-assay variation was determined by calculating the coefficient of variation (CV) of three replicates within the same run, and the CVs obtained varied from 0.22% to 2.93% for different concentrations of standard plasmid and from 0.04% to 0.93% for different concentrations of bacterial DNA. The Ct values from three different runs were used to calculate the CV of the inter-assay, and the obtained CVs varied from 0.03% to 1.86% for different concentrations of plasmid and 0.15%–2.91% for different concentrations of bacterial DNA. The data indicated a high reliability of the multiplex qRT-PCR assay.

**TABLE 2:** Detection variability of the multiplex qRT-PCR assay

Variability	Materials	Concentration	*gltA*		*rmpA/2*		*iucA*		KPC	
			Ct ± SD^*a*^	CV%	Ct ± SD	CV%	Ct ± SD	CV%	Ct ± SD	CV%
Intra-assay	Genome (CFU/mL)	10^8^	19.84 ± 0.13	0.66	20.3 ± 0.16	0.79	19.3 ± 0.18	0.93	19.42 ± 0.14	0.72
		10^6^	26.03 ± 0.10	0.38	26.7 ± 0.11	0.04	25.56 ± 0.06	0.23	25.8 ± 0.03	0.12
		10^4^	31.9 ± 0.16	0.50	32.78 ± 0.2	0.61	31.95 ± 0.21	0.66	32.31 ± 0.28	0.87
	Plasmid (copies/μL)	10^5^	21.05 ± 0.07	0.33	20.21 ± 0.08	0.40	21.04 ± 0.12	0.57	21.26 ± 0.14	0.66
		10^3^	27.75 ± 0.06	0.22	26.62 ± 0.78	2.93	27.54 ± 0.65	2.36	28.12 ± 0.13	0.46
		10^1^	33.59 ± 0.42	1.25	33.04 ± 0.29	0.88	33.86 ± 0.41	1.21	34.14 ± 0.36	1.05
Inter-assay	Genome (CFU/mL)	10^8^	19.73 ± 0.16	0.81	20.48 ± 0.25	1.22	19.69 ± 0.48	2.44	19.48 ± 0.08	0.41
		10^6^	25.97 ± 0.08	0.31	26.85 ± 0.21	0.78	25.91 ± 0.49	1.89	25.83 ± 0.04	0.15
		10^4^	32.29 ± 0.71	2.20	33.28 ± 0.71	2.13	32.63 ± 0.95	2.91	32.58 ± 0.38	1.17
	Plasmid (copies/μL)	10^5^	21.14 ± 0.12	0.57	20.21 ± 0.01	0.03	21.18 ± 0.2	0.94	21.45 ± 0.26	1.21
		10^3^	27.94 ± 0.28	1.0	26.89 ± 0.38	1.41	27.91 ± 0.52	1.86	28.36 ± 0.33	1.16
		10^1^	33.68 ± 0.12	0.36	32.98 ± 0.09	0.27	33.92 ± 0.08	0.23	34.18 ± 0.06	0.18

^*a*^ SD: Standard deviation.

### Detection of the KP in clinical isolates

A total of 84 Kp positive clinical samples were collected and used for the validation of the multiplex qRT-PCR assay, and 67 Kp strains were successfully isolated for antimicrobial susceptibility test and string test. Based on our multiplex qRT-PCR assay, a total of 31 hvKp strains and 53 cKp strains were identified. Among the 31 kvKp strains, *iucA*, *rmpA*, *rmpA2*, *iroB*, and *peg344* genes were detected in 31, 20, 18, 17, and 1 hvKp strains, respectively. The combination of *icuA/iroB*, *icuA/rmpA/iroB*, *iucA/rmpA/rmpA2*, *iucA/rmpA/rmpA2/iroB*, and *iucA/rmpA/rmpA2/iroB/peg344* were found in 5, 2, 8, 10, and 1 strains, respectively. Capsular serotyping analysis of the identified 31 kvKp strains showed that 5, 4, and 1 strains were K1, K2, and K5, respectively, with 21 strains unidentified. The 67 successfully isolated Kp strains were subjected to the string test, and 20 strains were positive and classified as hvKp strains. Of note, 26 discrepant samples between multiplex qRT-PCR and string test were found, with 16 strains from the qPCR identified hvKp and 10 strains from the string test identified hvKp. For the 10 samples identified as hvKp strains by the string test, no virulence-associated factors were detected by conventional PCR. Furthermore, nine KPC-positive strains (eight kvKp strains and one cKp strain) were identified by our multiplex qRT-PCR assay, and eight of them were successfully isolated and confirmed by the antimicrobial susceptibility test (Table S3).

### Validation of our multiplex qRT-PCR assay using outbred murine infection model

**Fig 3:**
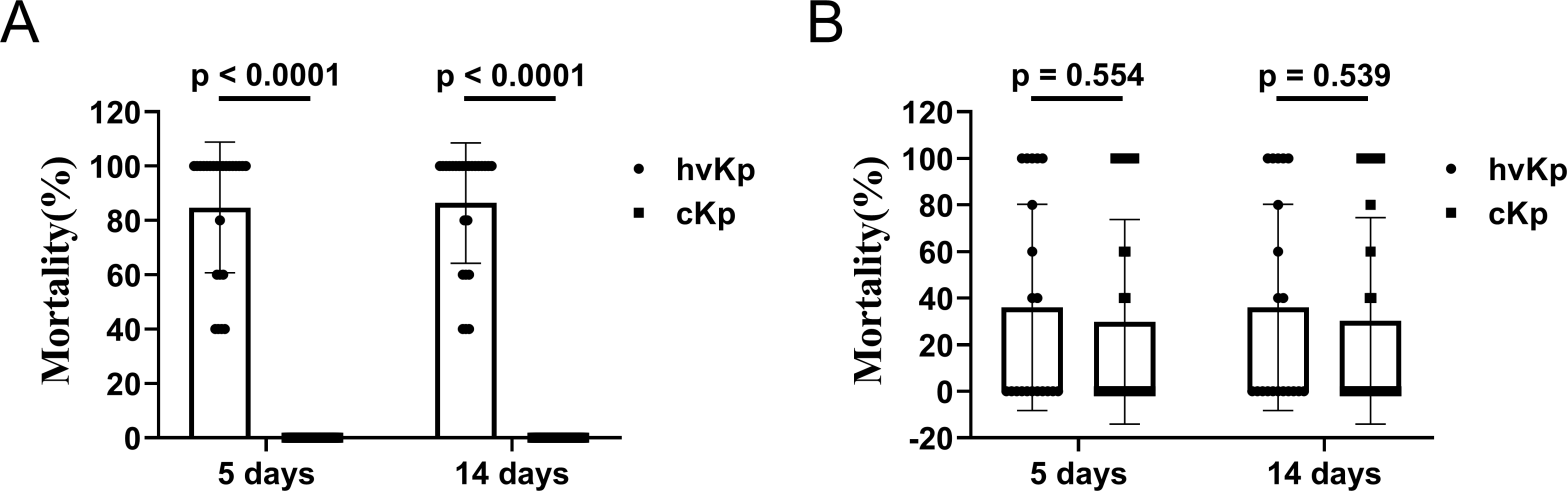
Comparative phenotype validation using outbred murine infection model. (**A**) The comparative mortality of CD1 mice infected with hvKP and cKP identified by the multiplex qRT-PCR assay. (**B**) The comparative mortality of CD1 mice infected with hvKP and cKP identified by the string test. The results were analyzed using the Mann-Whitney *U* test. A *P* value of <0.05 indicates statistical significance. *P < 0.05; **P < 0.001; and ***P < 0.001.

To evaluate the accuracy of the multiplex qRT-PCR, outbred murine infection model was used to evaluate the virulence of the Kp strains (27). Among the tested 67 Kp strains, 26 and 20 strains were identified as hvKp by the multiplex qRT-PCR assay and the string test, respectively. With the classification of multiplex qRT-PCR assay, the mortality rates of 5 and 14 days for the identified cKp strains were all 0%. For the identified hvKp strains, the mortality rates of 5 days were 40%, 60%, and 100% for 4, 3, and 18 strains, respectively, and the mortality rates of 14 days were 40%, 60%, 80%, and 100% for 3, 3, 1, and 18 strains, respectively. There were significant statistical differences between the mortality rates of the qRT-PCR-identified cKp and hvKp strains (*P* < 0.0001) (Fig. 3A). However, for the classification of the string test, both the mortality rates of 5 and 14 days showed no statistical differences between the identified cKp and hvKp strains (*P* = 0.554 and *P* = 0.539) (Fig. 3B).

## DISCUSSION

According to the data from China Antimicrobial Surveillance Network in 2023 (www.chinets.com), the overall infection rate of Kp has reached 14.06%, ranking the second in clinical pathogenic bacteria infection. What is worse, hvKp and CR-Kp have become two distinct evolutionary directions, resulting in high-risk Kp lineages (31). Therefore, increasing hvKp, CR-Kp, and even CR-hvKp strains have been recently identified (24), bringing great challenges to clinical treatment and public health. Early detection of hvKp and CR-Kp is of great significance for guiding the clinical treatment, prevention, and control of hospital infection. The conservative clinical definition of hvKp infection requires the occurrence of community-acquired and tissue-invasive infection in other healthy hosts (1). As hypermucoviscous is believed to be a distinctive phenotype of hvKp, a positive string test is frequently used, which is defined as a viscous string >5 mm in length formed by stretching bacterial colonies on an agar plate (25). Current reports of putative hvKp infection mainly rely on a positive string test and/or clinical features (26). However, this definition precludes recognition of hvKp infection in immunocompromised patients, patients with comorbidities, or patients in a healthcare setting. Meanwhile, studies have also found that not all hvKp strains are hypermucoviscous (32). Recently, one study has shown that >30 µg/mL of total siderophore concentration was strongly predictive of a kvKp isolate with a diagnostic accuracy of 0.96 (8). As the hypervirulence of hvKp strains is considered to be mediated by genes on a large virulence plasmid (8, 33, 34), *iroB*, *iucA*, *peg-344*, *rmpA*, and *rmpA2* genes were identified as biomarkers for hvKp with a diagnostic accuracy of over 0.95 (8). On this basis, another study has established a multiplex PCR method to distinguish hvKp from cKp (28). However, one recent study found that an extensively drug resistant (XDR) cKp strain was hypervirulent despite the absence of *iro*, *peg-344*, and *rmpA* being deleted in the acquired hvKp plasmid (33). These results support that *iucA* and/or either *rmpA* or *rmpA2* may be the most accurate and durable molecular biomarkers for hvKp (1). Our newly developed multiplex qRT-PCR contains the three genes of *iucA*, *rmpA*, and *rmpA2* with high sensitivity and specificity, which provides a rapid, sensitive, and specific assay for the identification of hvKp. For the clinical validation, the identified hvKp strains of our qRT-PCR assay were further confirmed by the prevalence of the virulence-associated genes using a previously established PCR assay (28). Notably, the *icuA* gene was found in all (31/31) the identified hvKp strains, which is consistent with previous findings that aerobactin is the dominant siderophore and a critical virulence factor for hvKp (9, 10). Moreover, *rmpA*, *rmpA2*, and *iroB* genes were lacking in some strains, while peg344 was found in only one strain, further supporting that *iucA* and/or either *rmpA* or *rmpA2* may be the most accurate and durable molecular biomarkers for hvKp (1).

Researchers have used *in vitro* neutrophil-mediated bactericidal assays and the *Galleria mellonella* infection model, either individually or in combination, to validate hvKp strains (27). Nevertheless, cKp strains may exhibit resistance to neutrophil-mediated killing, and existing data indicate that the *G. mellonella* model may not be a reliable tool for the precise discrimination of hvKp (27). Recent studies have found that the outbred murine infection model could distinguish hvKp and cKp with high accuracy (27, 35 – 37). Therefore, we use the model to validate our qRT-PCR assay. Our results from the infection model showed that over 40% mortality rates in our qRT-PCR identified hvKp strains, while no death was found in the identified cKp strains. For the string test, no statistical differences were found between the identified hvKp and cKp strains. These results further indicated that our multiplex qRT-PCR assay could accurately distinguish hvKp and cKp strains with far higher accuracy than the string test.

Since the first description in 1996, KPC enzymes have spread across countries and continents. The World Health Organization has classified CR-Kp as one of the critical priority pathogens requiring urgent research and development of new and effective antibiotic therapies (38). Conventionally, the activity of KPC enzymes can be detected by the Hodge test, acidimetric tests, and matrix-assisted laser desorption/ionization-time of flight (29, 30). However, high-level AmpC can give false positive results, particularly in Hodge (clover leaf) plates (18, 39). The definitive identification of carbapenemases in clinical isolates is best achieved by PCR of the corresponding genes (8, 18). Therefore, to identify KPC-mediated CR-Kp, we designed and validated a universal set of primers and probes targeting the conserved region of KPC genes that could recognize all the variants of KPC with high sensitivity. Most importantly, our assay can be performed within 2 hours, which strikingly shortened the turnaround time when compared with the antimicrobial susceptibility test (40). Moreover, an estimated 2.1 million serious infections were attributable to CR-Kp worldwide in 2014 alone (41). In 21 studies, the pooled mortality rate (*n* = 1,414) of KPC-producing CR-Kp isolates was 32.8% (95% CI: 27.7%–38.1%; *I* ^2^ = 61.3%) (19). The rapid identification of antimicrobial susceptibility can provide useful information for the targeted antimicrobial therapy, as a triple drug combination of colistin, tigecycline, and imipenem has been shown to correlate with improved survival for patients with bacteremia recently (18).

Despite the compelling nature of these findings, our assay has some limitations. First, our assay mainly detects the most common β-lactamase genotype of KPC, while other enzyme-producing genotypes, including GES, NDM, IMP, VIM, and OXA, are not covered (42 – 44). Second, our assay could not distinguish different KPC variants, thereby failing to convey epidemiological information and evolutionary significance of KPC. Sanger sequencing and multiplex qRT-PCR may help us to achieve this level of details (45, 46). Third, although the outbred murine infection may serve as the most accurate method to distinguish hvKp and cKp currently, it may not perfectly replicate human infections. Moreover, due to the limited number of clinical strains tested, validation of our assay with more clinical samples and some alternative or complementary methods is also needed.

In summary, we developed and validated a sensitive, rapid, single-reaction, and multiplex qRT-PCR assay for the simultaneous identification of hvKp and CR-Kp with excellent performance in sensitivity, specificity, and reliability. The multiplex qRT-PCR assay enables the rapid and accurate recognition of hvKp and CR-Kp in the clinical setting. This will allow early initiation of specific treatments depending on the phenotype of Kp strains, and fulfill the need for epidemiological surveillance studies on the prevalence of hvKp and CR-Kp in various populations.

## MATERIALS AND METHODS

### Nucleic acid extraction

Bacteria (*K. pneumoniae*, *S. pneumoniae*, *S. aureus*, *L. pneumophilia*, *H. influenzae*, *P. aeruginosa*, *A. baumannii*, *M. catarrhalis*, and *M. tuberculosis*), influenza A virus (panH1N1, H3N2, and H9N2), influenza B virus (Victoria and Yamagata lineages), human adenoviruses (HAdV-3, HAdV-7, and HAdV-55), human coronaviruses (HCoV-NL63, HCoV-229E, HCoV-OC43, and SARS-CoV-2) used in this study were isolated from clinical samples by our group. The cKp (SZKL-PMI-001), CR-cKp (SZKL-PMI-002), hvKp (SZKL-PMI-003), and CR-hvKp (SZKL-PMI-004) strains used to identify the LOD were confirmed by the outbred murine infection model, string test, antimicrobial susceptibility test, and conventional PCR (Table S2). DNA and RNA genomes were extracted using a TIANamp Bacteria DNA Kit (Tiangen Biotech, Beijing, China) and QIAamp RNA Viral Kit (Qiagen, Heiden, Germany) according to the manufacturer's protocol.

### Primer and probe design

The highly conserved *gltA* gene was selected for the detection of Kp. The aerobactin siderophore biosynthesis gene (*iucA*) and the capsular polysaccharide (CPS) regulator genes *rmpA* and *rmpA2* were selected as the specific molecular markers for hvKp, and the KPC gene as the molecular marker for CR-Kp. These gene sequences were downloaded from the GenBank database and aligned using the MEGA 7.0 software. Primer Premier 5 software was then used to design the primers and probes. The probes were labeled with four different fluorescent reporter dyes including FAM, ROX, VIC, and CY5 at the 5′-end, and the fluorescent quencher dye BHQ at the 3′-end. The sequences of the primer and probe sets are shown in Table 1.

### Detection of virulence-associated features and CPS genotyping

String tests for hypermucoviscosity were conducted as previously described (37). Briefly, all isolates were cultured on blood agar plates and incubated overnight at 37°C. After the incubation, an inoculating loop was used to touch the colonies gently and then lifted. A positive test was defined as a mucoid string >5 mm in length observed visually. Capsular serotyping of K serotype-specific alleles for K1, K2, K5, K20, K54, K57, and detection of the virulence-associated factors *iucA*, *rmpA*, *rmpA2*, *iroB*, and *peg344* were performed as previously reported (47). A 2× *TransTaq®*-T PCR SuperMix (+dye) (TransGen Biotech, Beijing, China) was used, and the reaction mixture was kept at 94°C for 2 min, followed by 35 cycles of 94°C for 30 s, 58°C for 30 s, 72°C for 2 min, and 72°C for 10 min. The PCR products were visualized and analyzed by agarose gel electrophoresis.

### Antimicrobial susceptibility test

Antimicrobial susceptibility to imipenem was determined by the agar dilution method according to the Clinical and Laboratory Standards Institute (CLSI) guidelines (document CLSI-M100-S30). Results were interpreted according to CLSI breakpoints. *P. aeruginosa* ATCC 27853 and *Escherichia coli* ATCC 25922 were used as controls for the antimicrobial susceptibility test.

### Outbred murine infection model

The outbred murine infection model has been described previously (27). In brief, CD1 mice (18–22 g; *n* = 5 per group) were challenged subcutaneously with challenge inocula of 1 × 10^7^ CFUs in 100 µL PBS for all 67 Kp strains. Animals were monitored for up to 14 days after challenge for the development of an in extremis state or death. The animal studies were reviewed and approved by the Laboratory Animal Ethics Committee of Guangdong Medical Laboratory Animal Center (C202107-11).

### Quantitative real-time PCR

The qRT-PCR assays were run on the ABI QuantStudio Dx Real-Time cycler (Applied Biosystems, Foster City, USA). Probe qRT-PCR Mix (Takara Bio, Dalian, China) was used as follows: 10 µL Probe qRT-PCR Mix, 0.4 µL forward primer (400 nM), 0.4 µL reverse primer (400 nM), 0.4 µL probe (400 nM), 0.2 µL RNase-free water, and 5 µL DNA samples. Optimal cycling conditions included an initial denaturation step of 95°C for 30 s, followed by 40 cycles of 95°C for 5 s (denaturation), and 60°C for 30 s (annealing and extension). Data were analyzed using the QuantStudio Real-Time PCR Software (Applied Biosystems). All samples were run in triplicate or three independent runs.

### Clinical samples

A total of 84 clinical samples from confirmed Kp-positive cases using commercial kits against Kp (Mabsky Biotech Co., Ltd., Shenzhen, China) were collected. Samples and case information are shown in Table S3. All the samples were subjected to the isolation of Kp strains, and a total of 67 Kp strains were successfully isolated, potentially due to low bacterial load or repeated freezing and thawing of some samples.

## Data Availability

The data supporting the findings of this study are available within the paper and the supplemental files. Source data are available on request from the corresponding author.

## SUPPLEMENTAL MATERIAL

Table S1 spectrum.00719-23-s0001.docx Specificity of the developed qRT-PCR.

Table S2 spectrum.00719-23-s0002.docx Detailed information of the four strains used for the determination of limit of detection.

Table S3 spectrum.00719-23-s0003.xls Detailed information of the clinical samples.

ASM does not own the copyrights to Supplemental Material that may be linked to, or accessed through, an article. The authors have granted ASM a non-exclusive, world-wide license to publish the Supplemental Material files. Please contact the corresponding author directly for reuse.
