# Supplementary material for: Development of a quadruple qRT-PCR assay for simultaneous identification of hypervirulent and carbapenem-resistant Klebsiella pneumoniae
Source: Microbiol Spectr. 2023 Dec 7;12(1):e00719-23. doi: 10.1128/spectrum.00719-23 (PMC10783029; doi:10.1128/spectrum.00719-23)
Supplement: Table S1 — Specificity of the developed qRT-PCR. [file spectrum.00719-23-s0001.docx]

**Table S1. Specificity of the developed qRT-PCR.**

| **Types and subtypes** | **Source/strains** | **Multiplex qPCR assays** | | | |
| --- | --- | --- | --- | --- | --- |
|  |  | **gltA** | **rmpA/2** | **iucA** | **KPC** |
| H3N2 | CAS0001 | U | U | U | U |
| H1N1 | CA04 | U | U | U | U |
| H9N2 | 04.15SZBAXQ005 | U | U | U | U |
| IBV | Victoria | U | U | U | U |
| IBV | Yamagata | U | U | U | U |
| HAdV-3 | Clinical isolate | U | U | U | U |
| HAdV-7 | Clinical isolate | U | U | U | U |
| HAdV-55 | Clinical isolate | U | U | U | U |
| HCoV-NL63 | Clinical isolate | U | U | U | U |
| HCoV-229E | Clinical isolate | U | U | U | U |
| HCoV-OC43 | Clinical isolate | U | U | U | U |
| SARS-CoV-2 | Clinical isolate | U | U | U | U |
| Streptococcus pneumoniae | Clinical isolate | U | U | U | U |
| Staphylococcus aureus | Clinical isolate | U | U | U | U |
| Legionella pneumophilia | Clinical isolate | U | U | U | U |
| Haemophilus influenzae | Clinical isolate | U | U | U | U |
| Pseudomonas aeruginosa | Clinical isolate | U | U | U | U |
| Acinetobacter baumannii | Clinical isolate | U | U | U | U |
| Moraxella catarrhalis | Clinical isolate | U | U | U | U |
| Mycobacterium tuberculosis | Clinical isolate | U | U | U | U |
| Klebsiella pneumoniae (cKp) | SZKL-PMI-001 | 14.52 | U | U | U |
| Klebsiella pneumoniae (CR-cKp) | SZKL-PMI-002 | 16.88 | U | U | 17.04 |
| Klebsiella pneumoniae (hvKp) | SZKL-PMI-003 | 15.21 | 14.71 | 15.06 | U |
| Klebsiella pneumoniae (CR-hvKp) | SZKL-PMI-004 | 13.68 | 14.11 | 14.26 | 15.24 |

**^a^** not available.

^b^ not applicable.

^c^ undetected.
