# Supplementary material for: Development of a quadruple qRT-PCR assay for simultaneous identification of hypervirulent and carbapenem-resistant Klebsiella pneumoniae
Source: Microbiol Spectr. 2023 Dec 7;12(1):e00719-23. doi: 10.1128/spectrum.00719-23 (PMC10783029; doi:10.1128/spectrum.00719-23)
Supplement: Table S2 — Detailed information of the four strains used for the determination of limit of detection. [file spectrum.00719-23-s0002.docx]

**Table S2. Detailed information of the four strains used for the determination of limit of detection.**

| **No.** | **Age** | **Sex** | **Department** | **Types of infection** | **Outcome** | **Sample Type** | **MIC (μg/ml)** | **Carbapenem-resistant** | **Stringing test** | **Conventional PCR** | | | | | **Mortality in CD1 murine infection model (%)** | |
| --- | --- | --- | --- | --- | --- | --- | --- | --- | --- | --- | --- | --- | --- | --- | --- | --- |
|  |  |  |  |  |  |  |  |  |  | **rmpA** | **rmpA2** | **iroB** | **iucA** | **peg344** | **5 days** | **14 days** |
| SZKL-PMI-001 | 70 | Female | ICU | Nosocomial | Discharged | Sputum | 1 | Negative | Negative | √ | √ | √ | √ | U | 100 | 100 |
| SZKL-PMI-002 | 26 | Male | ICU | Community acquired | Died | Bronchoalveolar lavage fluid | 0.5 | Negative | Negative | U | U | U | U | U | 0 | 0 |
| SZKL-PMI-003 | 62 | Male | Infectious disease department | Community acquired | Died | Bronchoalveolar lavage fluid | ≥ 8 | Positive | Positive | U | U | U | U | U | 0 | 0 |
| SZKL-PMI-004 | 72 | Female | ICU | Nosocomial | Died | Sputum | ≥ 8 | Positive | Positive | √ | √ | U | √ | U | 100 | 100 |

MIC: Minimal inhibitory concentration.

ICU: Intensive care unit.

U: Undetected.
